# Clinical Characterization of Data-Driven Diabetes Clusters of Pediatric Type 2 Diabetes

**DOI:** 10.1155/2023/6955723

**Published:** 2023-07-18

**Authors:** Mahsan Abbasi, Mustafa Tosur, Marcela Astudillo, Ahmad Refaey, Ashutosh Sabharwal, Maria J. Redondo

**Affiliations:** ^1^Electrical and Computer Engineering, Rice University, Houston, TX, USA; ^2^Department of Pediatrics, Division of Diabetes and Endocrinology, Baylor College of Medicine, Texas Children's Hospital, Houston, TX, USA; ^3^Children's Nutrition Research Center, USDA/ARS, Houston, TX, USA

## Abstract

**Background:**

Pediatric Type 2 diabetes (T2D) is highly heterogeneous. Previous reports on adult-onset diabetes demonstrated the existence of diabetes clusters. Therefore, we set out to identify unique diabetes subgroups with distinct characteristics among youth with T2D using commonly available demographic, clinical, and biochemical data.

**Methods:**

We performed data-driven cluster analysis (K-prototypes clustering) to characterize diabetes subtypes in pediatrics using a dataset with 722 children and adolescents with autoantibody-negative T2D. The six variables included in our analysis were sex, race/ethnicity, age, BMI *Z*-score and hemoglobin A1c at the time of diagnosis, and non-HDL cholesterol within first year of diagnosis.

**Results:**

We identified five distinct clusters of pediatric T2D, with different features, treatment regimens and risk of diabetes complications: Cluster 1 was characterized by higher A1c; Cluster 2, by higher non-HDL; Cluster 3, by lower age at diagnosis and lower A1c; Cluster 4, by lower BMI and higher A1c; and Cluster 5, by lower A1c and higher age. Youth in Cluster 1 had the highest rate of diabetic ketoacidosis (DKA) (*p* = 0.0001) and were most prescribed metformin (*p* = 0.06). Those in Cluster 2 were most prone to polycystic ovarian syndrome (*p* = 0.001). Younger individuals with lowest family history of diabetes were least frequently diagnosed with diabetic ketoacidosis (*p* = 0.001) and microalbuminuria (*p* = 0.06). Low-BMI individuals with higher A1c had the lowest prevalence of acanthosis nigricans (*p* = 0.0003) and hypertension (*p* = 0.03).

**Conclusions:**

Utilizing clinical measures gathered at the time of diabetes diagnosis can be used to identify subgroups of pediatric T2D with prognostic value. Consequently, this advancement contributes to the progression and wider implementation of precision medicine in diabetes management.

## 1. Introduction

Type 2 diabetes (T2D) is the most common form of diabetes in adults. More than 37 million Americans have diabetes (about 1 in 10), and approximately 90%–95% of them have T2D [1]. The apparent clinical and genetic complexity of patients with T2D suggest a tremendous pathophysiological heterogeneity [2]. Not all individuals with T2D fit easily into a single class and the current traditional symptom-based definition of T2D can be refined into additional subclassifications [3–10]. Further classifying T2D into subgroups may permit us to understand differences in etiology and pathogenesis, observe differential risk for complications, and improve existing treatment regimens to be more targeted to the characteristics in each individual.

Several recent studies have attempted to deconstruct the heterogeneity of T2D using biomarkers and phenotypic data to identify T2D subgroups. A Swedish cohort identified five replicable clusters of adults with diabetes with different patient characteristics and risk of diabetic complications [3]. Several studies demonstrated improved assessment of cardiovascular risks when subgrouping adults with T2D [9, 10]. These clusters have been replicated in many other adult populations [4–6]. The addition of genetic information allowed further refinement of the clusters [7, 8]. A variety of approaches have been used for an optimal subclassification in different settings (e.g., clinical practice versus research and rich versus limited resources).

However, it remains unknown whether phenotypic data and clustering approaches used previously in adult-onset diabetes can be applied to pediatrics. Pediatric T2D is increasing at a rapid pace particularly among racial and ethnic minorities [11, 12]. A disease previously considered to occur only in adult age is now observed in adolescents and even prepubertal children [13]. T2D has worse clinical course and health outcomes in youth than in adults. Youth with T2D have higher rate of complications and more frequent comorbidities than adults with T2D [14]. Moreover, compared with adolescents and young adults with Type 1 diabetes (T1D), youth with T2D have increased risk for cardiovascular complications including macrovascular disease, with resultant decreased life expectancy [15, 16]. Dissecting the heterogeneity of T2D in youth may help tailor treatments and ultimately improve prognosis.

We hypothesized that a data-driven analysis of clinical data in a pediatric population with T2D could identify distinct T2D subtypes. Therefore, we set out to perform data-driven cluster analysis to identify unique diabetes subgroups with distinct characteristics among youth with T2D. By identifying a more precise characterization of recently diagnosed pediatric T2D, this will bring us closer to the goal of tailoring treatment strategies to the individual and mitigating the risk of diabetes complications.

## 2. Methods

### 2.1. Study Population

Our dataset includes 722 children and adolescents (<19 years of age) with a clinical diagnosis of autoantibody-negative T2D treated at Texas Children Hospital between July 2016 and July 2019. We collected comprehensive data encompassing demographics, anthropometric measures, as well as clinical and laboratory information. The latter included hemoglobin A1c (A1c) levels and nonfasting, simultaneous measurements of serum glucose and C-peptide at the time of diagnosis, as well as lipid profile measures obtained within a year after diagnosis. We also collected BMI at diagnosis and sexual maturation (Tanner) staging documented by a pediatric endocrinologist within 3 months of the diabetes diagnosis when available. The presence of complications until their last follow-up visit (with mean follow-up duration of 2.7 years) was recorded. This study was approved, and informed consent requirement was waived by the Baylor College of Medicine Institutional Review Board.

### 2.2. Statistical Analysis

Since our goal was to identify clusters that have clinical relevance and reflect the underlying pathogenesis of T2D, we selected variables based on (i) widespread availability at the time of diagnosis or, for lipid panel, within the first year of diagnosis; (ii) previously reported prognostic value in predicting T2D development and/or progression; (iii) feature importance calculated by the random forest algorithm to find the variables which most drive the cluster assignment (see Figure S1 for details). Based on the above three driving characteristics, the resulting variables were age, sex, race/ethnicity, A1c, BMI *Z*-score at diagnosis, and non-HDL cholesterol. Age, BMI, sex, race/ethnicity, and A1c are well-known T2D predictors and prognostic factors, and modify clinical presentation [13, 17–20]. A1c is routinely measured in the clinical setting and outperforms fasting glucose for prediction of complications [21]. We chose non-HDL cholesterol to be included in the model since it is considered to be an important cardiovascular disease risk predictor in patients with diabetes [22] and is reliable when measured in both fasting and nonfasting states [23].

We used multivariate iterative imputation method [24–26] to estimate 12% missing values of incomplete features (BMI at diagnosis and non-HDL) by modeling them as a function of other features such as BMI at Visit 1, LDL, and triglyceride.

Since the selected features consisted of both numerical and categorical variables, we used K-prototypes clustering to accommodate for all features. This algorithm offers the advantage of working with mixed data types. It measures distance between numerical features using Euclidean distance (like K-means) but also measures the distance between categorical features using the number of matching categories [27, 28]. Cluster analysis was performed on values scaled to a mean value of 0 and a standard deviation (SD) of 1. The silhouette analysis indicated that there are five clusters.

Continuous values are expressed as median (first quartile–third quartile) because all the variables are nonparametric. We applied Mann–Whitney *U* test to compare continuous characteristics and factors between two groups (e.g., sex) and Kruskal–Wallis test for comparing among multiple groups (e.g., between diabetes subgroups). Chi-Square (*χ*^2^) test was used to assess distribution of categorical outcomes (e.g., diabetes complications) among clusters. Statistical analysis was considered significant if two-sided *p*-values were <0.05. All analyses were conducted using Python.

## 3. Results

Of 722 patients in our dataset, 62.4% were females and 73.5% were obese. Median age at diagnosis was 13.7 years. The racial/ethnic distribution was 58.3% Hispanic, 29.4% non-Hispanic Black, 9.2% non-Hispanic White, 3% Asian, and 0.1% other races. The remaining characteristics are summarized in Table 1.

In clustering analysis, of the 722 individuals with diabetes, we excluded 273 participants missing many clinical measures and laboratory data.

Since BMI at diagnosis and BMI at Visit 1 were highly correlated (*r* = 0.93, *p* = 0.0001), and we had 12.4% of BMI values missing at diagnosis, we imputed the missing values for BMI at diagnosis using the collected BMI measures at Visit 1. Similarly, because of the higher proportion of missing values for non-HDL (11.8%), we modeled it as a function of other variables (LDL, HDL, and triglyceride) and estimated the missing values using the multivariate iterative imputation algorithm [24–26].

Eventually, we included 449 children with all six T2D-associated selected variables available based on the prerequisite of the clustering model.

### 3.1. Cluster Distribution and Characteristics at Baseline

**Figure 1:**
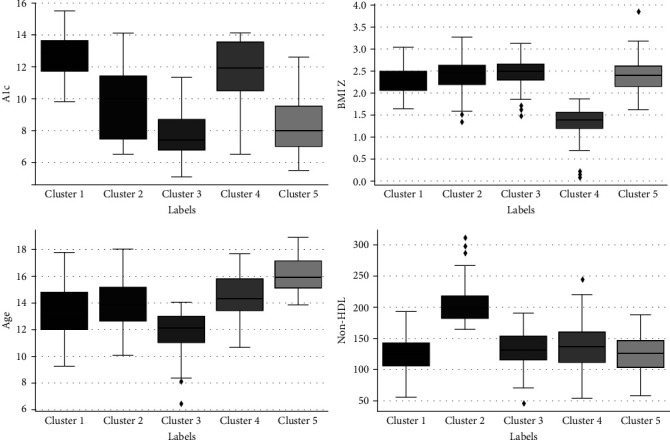
The distribution of numerical variables in the clusters.

Using K-prototypes clustering, five subgroups were identified. The frequency and distribution of the five clusters obtained by K-prototypes clustering are shown in Table 2 and Figure 1. We labeled the clusters based on their most distinctive characteristics.

We used feature importance (Figure S2) to understand the characteristics of each cluster. Using feature importance analysis, we labeled the clusters based on the most prominent features contributing to forming that cluster and the distribution of those features (see Figure S1 for details). Each cluster is described below:

1. Cluster 1 (*n* = 102 individuals) was characterized by higher A1c (>10%)
2. Cluster 2, (*n* = 48 individuals) was characterized by higher non-HDL (>160 mg/dL)
3. Cluster 3, (*n* = 131 individuals) was characterized by lower age (<14 years), lower A1c (<10%)
4. Cluster 4, (*n* = 49 individuals) was characterized by lower BMI (*Z*-score <2), higher A1c (>10%)
5. Cluster 5, (*n* = 119 individuals) was characterized by higher age at diagnosis (>14 years), and lower A1c (<10%).

### 3.2. Diabetes Complications and Treatments in Clusters

After identifying the five diabetes subgroups, we assessed whether they were differentially associated with race/ethnicity, levels of systolic and diastolic blood pressures, and prevalence of microalbuminuria, acanthosis nigricans (AN), diabetic ketoacidosis (DKA), chronic kidney disease (CKD), nonalcoholic fatty liver disease (NAFLD), polycystic ovarian syndrome (PCOS), hypertension, thyroid disease, retinopathy, and prescription of insulin and metformin.

**Figure 2:**
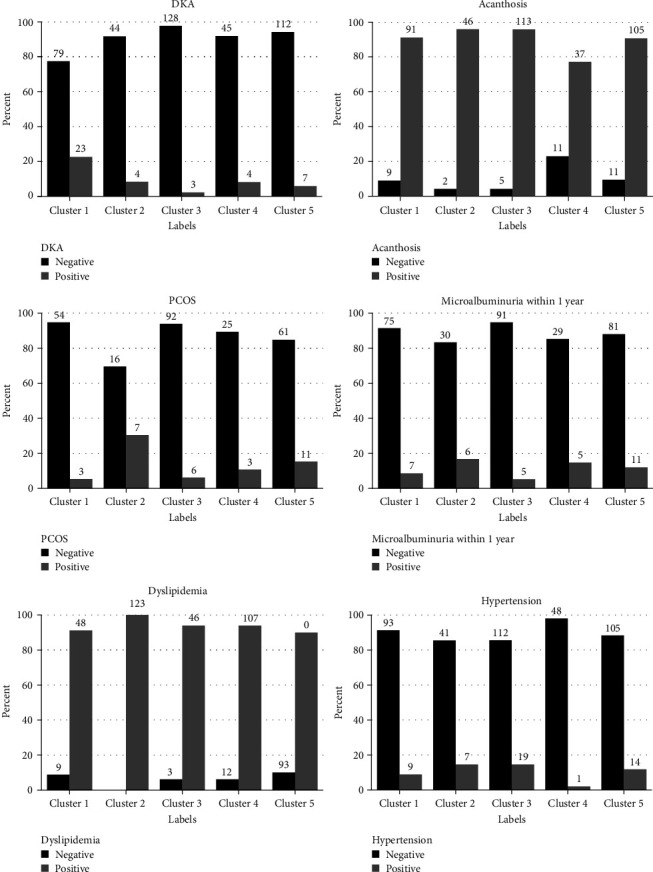
The distribution of selected characteristics and diabetes complications in each cluster.

We observed different risks for complications in each group based on our clustering model (Figure 2) and examined the associations between clusters with certain traits and prevalence of complications in those clusters.

**Figure 3:**
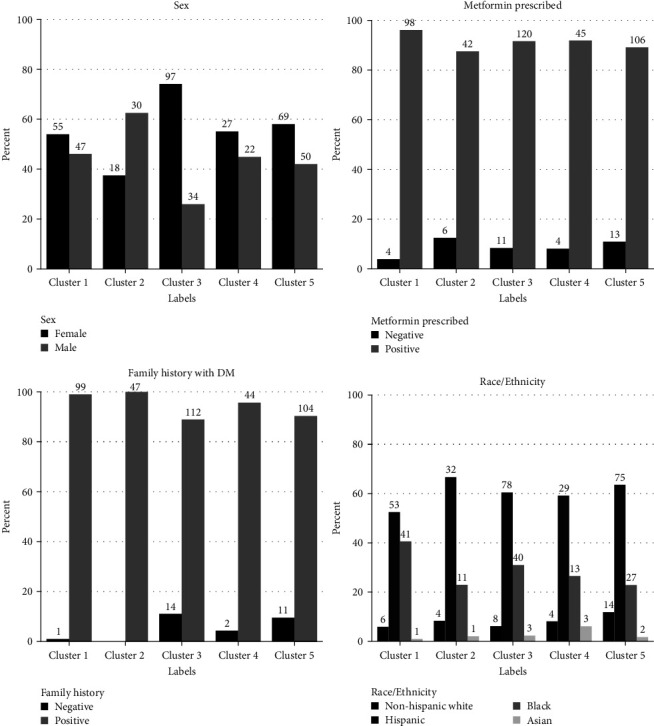
The distribution of sex, treatments, race/ethnicity, and family history of diabetes in each cluster.

In support of the clustering, we further explored different distribution of sex, treatment regimens, race/ethnicity, and family history of diabetes among subgroups (Figure 3).

Overall, we found that individuals in Cluster 1 (i.e., those with higher A1c) had the highest risk of DKA (*p* < 0.0001) and were most likely to get prescribed metformin (96% prescribed, *p* = 0.06).

Conversely, individuals with higher non-HDL levels (100% positive dyslipidemia in Cluster 2, *p* = 0.04) were more prone to PCOS (*p* = 0.001).

One out of eight individuals in Cluster 3 (12.5%) did not have any relative diagnosed with diabetes mellitus (*p* = 0.01), and they were not prone to DKA (*p* = 0.001) and microalbuminuria (*p* = 0.06). Furthermore, almost one in three individuals (30%) in Cluster 4, with lower BMI and higher A1c, did not have AN documented in their chart within 3 months of T2D diagnosis (*p* = 0.0003) and they were least frequently diagnosed with hypertension (*p* = 0.03).

Furthermore, Cluster 2 mostly consist of males at birth (*p* = 0.01) compared to Cluster 3 with mostly female (*p* < 0.0001).

We observed that several other ethnic trends were statistically significant and merit further analysis with larger datasets. For example, while we observed that the cluster with higher A1c (Cluster 1) was the least common subgroup assignment for Hispanics and the most common subgroup assignment for African Americans (*p* = 0.02). Cluster 5 with lower A1c and higher age was the most common subgroup for non-Hispanic Whites (*p* = 0.01).

In our secondary analysis, we verified the stability of the clustered subgroups and their features by excluding non-HDL from the clustering variables and conducting the analysis on 540 individuals with the available data at the time of diagnosis (A1c, age, BMI at diagnosis, and sex). Using K-prototypes clustering, four clusters were detected with the same characteristics as described above except for the higher non-HDL cluster.

1. Cluster 1 (*n* = 149 individuals) was characterized by higher A1c with higher risk of DKA (*p* < 0.0001). All individuals in this cluster had A1c above 10%.
2. Cluster 2, (*n* = 78 individuals) was characterized by lower BMI with the lowest rate of AN (*p* = 0.0006) and hypertension (*p* = 0.009). All individuals in this clusters had BMI *Z*-score under Cluster 2.
3. Cluster 3, (*n* = 160 individuals) was characterized by higher age at diagnosis and lower A1c. All individuals in this cluster were older than 14 years of age at diagnosis and had A1c under 11%.
4. Cluster 4, (*n* = 153 individuals) was characterized by lower age at diagnosis, and lower A1c with least frequent DKA diagnosis (*p* = 0.001) and lowest history of diabetes in family (0.0007). All individuals in this cluster had A1c under 11% and were younger than 14 years old at diagnosis.

## 4. Discussion

In this study, we discovered five distinct phenotypic clusters of pediatric T2D using widely available variables at the time of diabetes diagnosis. Furthermore, our findings show that the prevalence of complications and treatment strategies can differ even at the time of diabetes diagnosis based on a more precise subclassification of pediatric T2D.

To our knowledge, this is the first study that defines clusters in pediatric T2D. A recent study in adults identified five unique diabetes subgroups qualitatively grouped by presence of autoimmune antibodies, insulin deficient, insulin resistant, obesity related, and age-related diabetes [3]. They found associations between the clusters and disease progression and some risks of diabetes complications. These results have been replicated in many other adult populations with diabetes and they all demonstrated improved assessment of cardiovascular risks when subgrouping adults with T2D. Consistent with those studies, we found that in pediatric T2D, it is also possible to define distinct clusters with different characteristics. T2D is commonly recognized as a heterogeneous disease with different disease-causing pathways and disorders of the insulin resistance syndrome [29], suggesting that it requires unique treatment strategies. Thus, subclassification may improve diabetes management.

In an effort to characterize unique subgroups of pediatric T2D, we applied a data-driven approach to identify five distinct phenotypes based on six widely available characteristics and then assessed whether the subgroups are differentially associated with risk factors for diabetes complications, associated conditions, and treatment strategies.

All individuals in some subgroups shared certain characteristics. For example, Cluster 2 consisted of individuals with non-HDL levels of 160 mg/dL or higher (with median of 197.5 mg/dL) and was labeled as the higher non-HDL group relative to the other clusters (with median of 128 mg/dL). Conversely, some subgroups were differentiated based on the most prominent characteristic shared by more than 95% of the individuals in that group. For instance, Cluster 4 primarily comprised individuals 14 years or older with A1c levels less than 10% (labeled as the higher age, lower A1c cluster), had only a few exceptions of 17-year-olds with the A1c level of 10.9 mg/dL. Same for Cluster 5, which consist of 95% individuals younger than 14 years of age with A1c levels less than 10% and thus, was labeled as the lower age, lower A1c cluster.

Assigning labels to the subgroups is a crucial step in comparing disease progression, treatment, and development of diabetic complications among clusters with certain traits.

In our analysis, youth in Cluster 1, with higher A1c and lower non-HDL, had the highest rate of DKA. This is in line with previous studies demonstrating that individuals with T2D and high A1c levels face greater risk for DKA, and thus, A1c could be a potential marker of DKA risk in T2D [30–32].

Additionally, the individuals in Cluster 1 had the highest rate of being prescribed metformin (*p* = 0.06) which is reliable evidence as to the widespread use of metformin to reduce A1c in T2D population [33] even among children. Of note, metformin may have a role in the development of lactic acidosis (LA) and hyperlactemia [34] in patients admitted with DKA. These findings generate the question of whether metformin, which continues to be widely prescribed as a first-line therapy for the treatment of diabetes [35], is the optimal treatment for youth in this cluster.

Cluster 2 was characterized by having higher non-HDL levels. This cluster had the highest rate of PCOS diagnosis which is supported by previous studies associating elevated non-HDL with PCOS women [36].

Another important finding in our study was a significant difference in the distribution of AN and hypertension diagnosis among clusters. The prevalence of AN was lowest among individuals with lower BMI and higher A1c (Cluster 4), suggesting that AN is closely associated with obesity as a manifestation of cutaneous insulin resistance [37–39], while it may also develop prior to the onset of obesity [40]. The higher levels of A1c in this subgroup brings up the hypothesis that AN might not be directly associated with A1c [41] and BMI might be a more important factor in developing this complication.

Moreover, the same cluster had the lowest rate of hypertension, similar to the findings of previous cross-sectional studies conducted on adults with T2D, reinforcing an association between hypertension and obesity in T2D [42, 43].

The prevalence of DKA and diagnosis of microalbuminuria was lowest among younger individuals with low A1c, which is the same subgroup with least frequent family history of diabetes. This finding suggests the hypothesis that having relatives with diabetes play an important role in the individual risks of developing some diabetes complications [44].

However, the association between genetic predispositions and the development of some diabetes-related disorders is still largely unknown [45]. Thus, further investigations are needed to illustrate the role of genetic susceptibility in diabetes complications and to provide important insight into the etiology of T2D.

Our study has several strengths. First, this is the application of a clustering algorithm that also works with categorical variables (sex, race, and ethnicity) to accommodate for those characteristics in our subclassification. In addition, we used a parsimonious number of clinical tests and biological information needed to categorize individuals into diabetes subgroups at the time of diabetes diagnosis.

Limitations of this study include a retrospective design and a relatively small sample size. Nevertheless, this is a single-center cohort of youth with T2D cared for at the largest pediatric hospital in the United States, in an area with elevated prevalence of obesity and rich racial and ethnic diversity. We used of a limited number of variables from demographic, clinical, and laboratory characteristics in the clustering model. However, this is the trade off of our goal to use variables that are typically available in youth with recent onset T2D in the clinical setting. Moreover, data for clustering characteristics, cardiovascular risk factors, and diabetes complications may be subject to change, dependent on diabetes duration. However, the fact that different clustering analysis led to similar results with the same subgroup characteristics, suggests that the clusters are stable at a given time. In addition, it is important to note that the variables used for clustering in this study were carefully selected from a wide range of available variables using feature importance analysis. This suggests that our model and its results are highly reliable. It is possible that future studies with larger sample sizes and the inclusion of additional data, such as genetics, could allow for many novel aspects, including finer classification of diabetes subgroups and potential sex and racial/ethnic differences among pediatric subgroups. Furthermore, describing the changes in the characteristics of clusters as diabetes duration increases will require longitudinal studies. In last, although the clusters that we describe may suggest new hypothesizes for distinct pathophysiological mechanisms, further studies are needed to understand etiology of subgroups.

In conclusion, the treatment regimens and risk stratifications can differ even at the time of diabetes diagnosis based on a more precise characterization of pediatric T2D. Intervention trials and novel treatment regimens tailored to subgroups may improve cardiovascular outcomes in children with T2D.

## Figures and Tables

**Table 1:** Baseline demographic, clinical, and biochemical characteristics of children with T2D.

	*N* (total number of participants with available data)	Median (first quartile to third quartile) or *n* (percent)
Age at diagnosis, years	722	13.7 (12–15.5)

Female, *n* (%)	722	451 (62.5%)

Race/ethnicity, *n* (%)	708	65 (9.2%) non-Hispanic White 413 (58.3%) Hispanic 208 (29.4%) non-Hispanic Black 21 (3%) Asian 1 (0.1%) Other

BMI (*Z*-score) at diagnosis	544	2.32 (2.0–2.6)

A1c at diagnosis, %	631	9.5 (7.2–11.8)

Non-HDL cholesterol, mg/dL	466	131 (109–158)

HDL cholesterol, mg/dL	466	37 (31–44)

C-peptide, ng/mL	470	3.0 (1.83–4.9)

*Note:* Numbers in all tables are median (first quartile–third quartile).

**Table 2:** Baseline characteristics of clustering-based subgroups.

Characteristic	Cluster 1: higher A1c	Cluster 2: higher non-HDL	Cluster 3: lower A1c, lower age	Cluster 4: lower BMI, higher A1c	Cluster 5: higher age, lower A1c	*p*-Value
*N* (%)	102 (22.7%)	48 (10.7%)	131 (29.2%)	49 (10.9%)	119 (26.5%)	
*N* female (%)	47 (58.8%)	23 (57.5%)	16 (41%)	32 (32.6%)	23 (23.2%)	
Age at diagnosis, years	13.4 (12.0–14.8)	13.8 (12.6–15.1)	12.1 (11.0–12.9)	14.3 (13.3–15.8)	15.9 (15.1–17.1)	<0.001
BMI *Z*-score at diagnosis	2.2 (2.1–2.5)	2.5 (2.2–2.6)	2.5 (2.3–2.6)	1.4 (1.2–1.6)	2.4 (2.2–2.6)	<0.001
A1c at diagnosis, %	12.8 (11.7–13.6)	10.0 (7.4–11.4)	7.4 (6.8–8.6)	11.9 (10.5–13.5)	8.0 (7.0–9.5)	<0.001
Non-HDL cholesterol, mg/dL	124.1 (104.2–142.5)	197.5 (181.2–217.2)	131.0 (115.0–153.5)	136.0 (111.0–160.0)	125.0 (102.0–146.1)	<0.001

*Note*: Numbers in all tables are median (first quartile–third quartile).

## Data Availability

The dataset used to support the findings of this study are available from the lead PI, Maria Jose Redondo (redondo@bcm.edu) upon request.

## Supplementary Materials

Supplementary Materials Figure S1: SHAP analysis showing the impact of the selected variables on the model. Figure S2: feature importance analysis showing the contribution of features on each cluster assignment.
