# Supplementary material for: Clinical Characterization of Data-Driven Diabetes Clusters of Pediatric Type 2 Diabetes
Source: Pediatr Diabetes. Author manuscript; Available in PMC 2024 May 1. (PMC11062019; doi:10.1155/2023/6955723)
Supplement: Supplementary Material [file NIHMS1957962-supplement-Supplementary_Material.docx]

**Appendix:**

Feature of Importance was analyzed using the random forest algorithm [46] to quantify the contribution that each feature brings to the clustering and understand the relationship between the selected set of variables and the output model. In other words, the contribution of a variable is calculated by the explanatory power that it brings to the final clustering model.


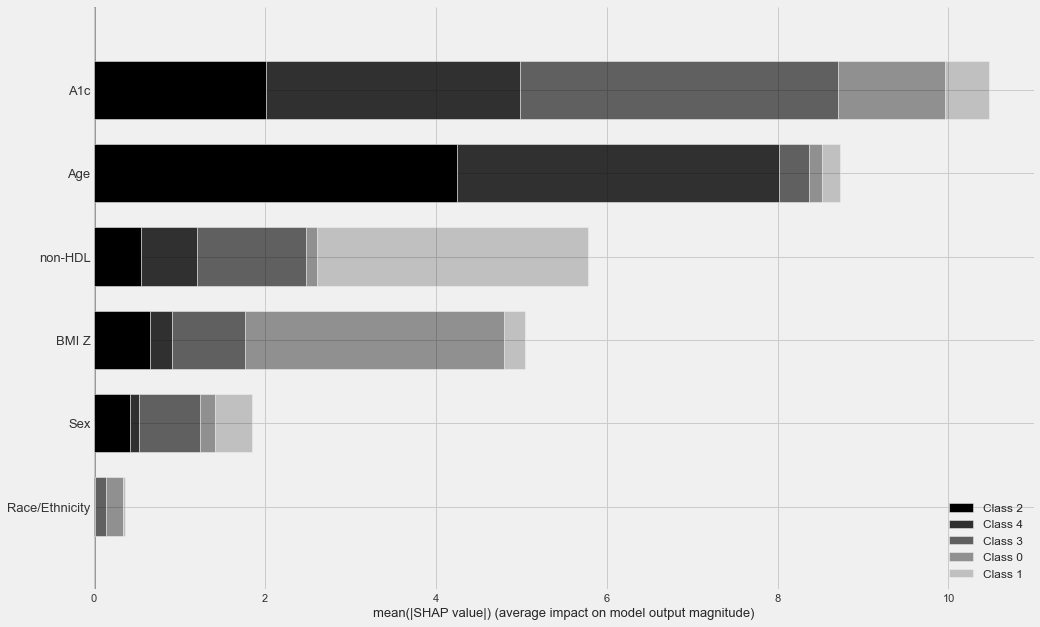


**Figure S1.** Feature importance showing the impacts of the selected variables on the model and ranking them by their importance. The colors show how much the variables contributed to modeling each cluster.

**Figure S1** shows the average impact in absolute terms (mean SHAP values) [47] of the features on the clustering model [48]. Based on the calculated feature importance (**Figure S1**) and the criteria described in the variable selection section, the following subset of variables were included in the model: A1c, BMI and age at the time of diabetes diagnosis; race/ethnicity; sex; and non-HDL within the first year of diabetes diagnosis.

As noted in the main text, the clusters were labeled based on the most contributing feature in forming that cluster.

We fit a random forest classification model [46] on the dataset using the cluster labels as the target variable and then performed SAPH analysis [47] to compute the contribution of each feature to the prediction. **Figure S2** shows the overall effect of the features on each cluster prediction.

We labeled the clusters based on the most prominent features contributing to each cluster assignment. For example, non-HDL have the most impact in forming cluster 2 (it explains more than 70% of that cluster assignment), hence cluster 2 is labeled based on non-HDL (higher non-HDL).


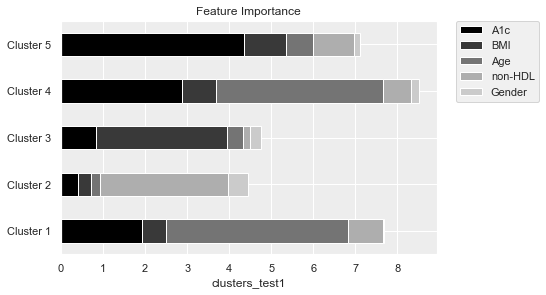


**Figure S2.** Feature importance showing the contribution of features on each cluster assignment. X axis quantifies how much the variables contributed to modeling each cluster.
